# Supplementary material for: Unraveling the liquid gliding on vibrating solid liquid interfaces with dynamic nanoslip enactment
Source: Nat Commun. 2022 Nov 3;13:6608. doi: 10.1038/s41467-022-34319-0 (PMC9633805; doi:10.1038/s41467-022-34319-0)
Supplement: Supplementary file 1 — Supplementary information [file 41467_2022_34319_MOESM1_ESM.pdf]

Unraveling the liquid gliding on vibrating solid liquid interfaces with dynamic nanoslip enactment

Amir Farokh Payam[1,2*†], Bogyoung Kim[3], Doojin Lee[3‡§] & Nikhil Bhalla[1,2¶||]

[1]*Nanotechnology and Integrated Bioengineering Centre (NIBEC), School of Engineering, Ulster University, Jordanstown, Shore Road, Northern Ireland, BT37 0QB, United Kingdom*

[2]*Healthcare Technology Hub, Ulster University, Jordanstown, Shore Road, Northern Ireland, BT37 0QB, United Kingdom*

[3]*Department of Polymer Science and Engineering, Chonnam National University, Gwangju, 61186, Republic of Korea*

Emails of corresponding authors:

[*]Corresponding author.
[†]E-mail: a.farokh-payam@ulster.ac.uk

[‡]Corresponding author.
[§]E-mail: dlee@chonnam.ac.kr

[¶]Corresponding author.
[||]E-mail: n.bhalla@ulster.ac.uk

1. Liquid slip model development

We use the small load approximation [1] to write the frequency shift and damping ratio in terms of the load impedance:

$$Z_L = \frac{-i\pi Z_Q(\Delta f + if\Delta\mathrm{D}/2)}{f_0}$$

(1)

Where $\Delta\mathrm{D}$ is damping rate, f_0 is the natural resonant frequency of the QCM in free state i.e. 10 MHz in this work, f is the frequency of the QCM under operation i.e. when exposed to fluid, Δf is frequency shift calculated as $f - f_0$, Z_L and Z_Q are the complex load impedance and quartz impedance respectively and are described by:

$$Z_Q = \sqrt{c_q \rho_q}$$

(2)

$$Z_L = \left[\frac{F_f}{v_s}\right]_{z=0}$$

(3)

Where c_q and ρ_q are the stiffness and density of quartz, respectively. v_s is the velocity phasor and F_f is the shear force applied on the film on the substrate per unit area.

In our model we consider three different cases: substrate–layer interface boundary conditions without and with slip; and substrate-layer interface boundary condition with slip and inertia force due to the contribution of inertia of the first water layer to the momentum/energy transfer at the liquid/solid interface. The schematic of the substrate-liquid interface is given in Fig. S1.

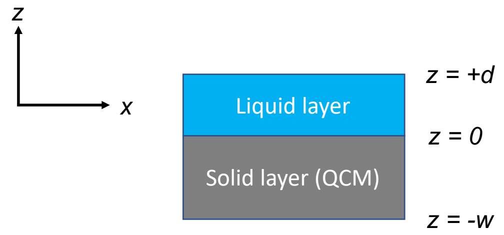

***Fig. S1.** Definition of axes for quartz crystal substrate with a liquid over layer.*

2. Oscillating Boundary Layer

In this section, the fluid system near a vibrating plane wall in the Stokes flow regime is discussed [1]. We use load impedance method which relates the shear stress to the substrate speed at the interface and perturbation expansion is adopted for analysis of resonant frequency of the unloaded substrate [2]. Additionally, we consider the fluid with density ρ_f, shear viscosity η_f which obeys the Navier–Stokes equation for a Newtonian liquid, assuming fluid continuity and incompressibility. The equation describing the velocity fields is:

$$\frac{\partial \widetilde{v_f}}{\partial t} = \frac{\eta_f}{\rho_f}\frac{\partial^2 \widetilde{v_f}}{\partial z^2} = i\omega\widetilde{v_f}$$

(4)

Solutions of this equation can be sought using velocity function which rely on phasors to represent the amplitudes of the steady state solution:

$$\tilde{v}_f(z,t) = Re\left(v_f(z)e^{i\omega t}\right)$$

(5)

with the complex value amplitude phasor as:

$$v_f(z) = A_f e^{-\alpha z} + B_f e^{\alpha z}$$

(6)

where $\alpha = (1-i)/\delta$ and $\delta = \sqrt{2\eta_f/\omega\rho_f}$ measures the depth of penetration of the oscillating flow with the assumption of wall oscillation with angular frequency of ω. Applying the boundary condition of vanishing shear stress at the upper free surfaces of the substrate–fluid layer system, then:

$$\eta_f\left(\frac{\partial v_f}{\partial z}\right)_{z=d\to\infty} = 0$$

(7)

Which gives:

$$v_f(z) = A_f e^{-\alpha z}$$

(8)

For the substrate displacement $\widetilde{u_s}$, the following wave equation is considered:

$$\frac{\partial^2 \tilde{u}_s}{\partial z^2} = -\frac{\omega^2}{c_q^2}\tilde{u}_s$$

(9)

solutions of this equation can be sought using velocity function which rely on phasors to represent the amplitudes of the steady state solution:

$$\tilde{u}_s(z,t) = Re\left(u_s(z)e^{i\omega t}\right)$$

(10)

with the complex value amplitude phasor as:

$$u_s(z) = A_s e^{-\alpha_s z} + B_s e^{\alpha_s z}$$

(11)

where α_s is given by $\alpha_s = i\omega/c_s$. Applying the boundary condition of vanishing shear stress at lower free surfaces of the substrate–fluid layer system, generates following equation:

$$\mu_f \left(\frac{\partial u_s}{\partial z}\right)_{z=-w} = 0$$

(12)

which gives:

$$B_s = A_s e^{2\alpha_s w}$$

(13)

Therefore, the substrate displacement can be described by:

$$u_s(z) = A_s\left(e^{-\alpha_s z} + e^{\alpha_s (z+2w)}\right)$$

(14)

2.1. No slip condition

In the no-slip condition the fluid velocity and substrate velocity should be equal at the boundary between the substrate and layer. Equivalently, the displacements can be matched and therefore using equations (8) and (14), considering $v_f(0) = i\omega u_s(0)$:

$$A_f = i\omega A_s(1 + e^{2\alpha_s W})$$

(15)

Now the impedance associated with the Stokes flow will be calculated as given by equation (3).

The shear force exerted by the film on the substrate per unit area is given by the shear stress:

$$F_f = -\eta_f \left(\frac{dv_f}{dz}\right)_{z=0}$$

(16)

Hence:

$$F_f = \alpha \eta_f A_f$$

(17)

On the other hand, the velocity is given by:

$$v_s = i\omega u_s|_{z=0} = i\omega A_s(1 + e^{2\alpha_s w})$$

(18)

Substituting equations (15), (17) and (18) in (3) gives:

$$Z_L^{ns} = \frac{\alpha \eta_f A_f}{i\omega A_s(1+e^{2\alpha_s w})} = \alpha \eta_f$$

(19)

Considering $\alpha = (1-i)/\delta$ and $\delta = \sqrt{2\eta_f/\omega\rho_f}$ we have:

$$Z_L^{ns} = (1-i)\sqrt{\frac{\rho_f\eta_f\omega}{2}}$$

(20)

2.2. Slip boundary condition

The relationship between the fluid layer velocity gradient extrapolated from the bulk and the slip length b is shown diagrammatically in Fig. S2.

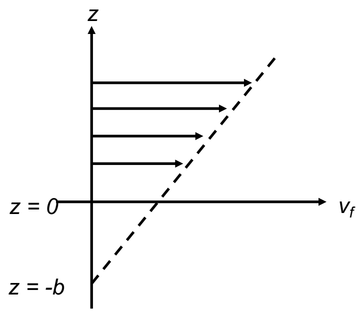

***Fig.S2**. Extrapolation of the fluid speed gradient from the bulk liquid and the relationship to the slip parameter b.*

In this case, applying the Ellis—Hayward [2], which adds the slip length to the no-slip velocity boundary condition, slip boundary condition is:

$$v_s(z=0) = i\omega u_s(z=0) = v_f(z=-b)$$

(21)

Therefore:

$$i\omega A_s(1+e^{2\alpha_s w}) = A_f e^{\alpha b}$$

(22)

which gives:

$$A_f = i\omega A_s e^{-\alpha b}(1+e^{2\alpha_s w})$$

(23)

Using equations (17), (18), (23) and (3) the complex impedance is obtained as:

$$Z_L^s = \frac{\alpha\eta_f A_f}{i\omega A_s(1+e^{2\alpha_s w})} = e^{-\alpha b}\alpha\eta_f = e^{-\alpha b}Z_L^{ns}$$

(24)

Using Taylor series and considering the first two terms of the series we have:

$$Z_L^s = (1+\alpha b)Z_L^{ns}$$

(25)

Hence:

$$\alpha b = \left(\frac{Z_L^s}{Z_L^{ns}} - 1\right)$$

(26)

Replacing $\alpha = (1 - i)/\delta$ and $\delta = \sqrt{2\eta_f/\omega\rho_f}$ in equation (26) gives:

$$\frac{b}{\delta} = (1 + i)\frac{(Z_L^s - Z_L^{ns})}{2Z_L^{ns}}$$

(27)

Equation (27) is a significant development, since it shows that the complex value represented by the physically meaningful dimensionless parameter $\frac{b}{\delta}$, which relates the slip length to the characteristic decay length of the fluid (penetration length).

As in the QCM, the frequency shift and damping rate are measured directly, using equations (1) and (20), the ratio between slip length and penetration length can be obtained from equation (27) and consequently slip length can be calculated.

2.3. Slip boundary condition with inertia force

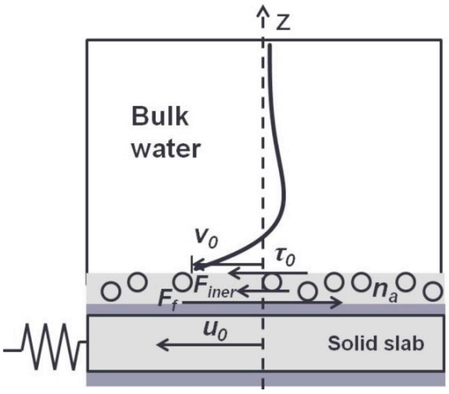

Fig. S3. *Schematic representation of the force balance on the interfacial liquid. This image is reprinted with permission from ref. 3. Copyright 2012 American Chemical Society.*

In this case, When the solid slab is loaded with liquid as shown schematically in Fig. S3., there will be a friction force per unit area exerted by the liquid on the solid surface which is the sum of shear stress of the liquid at the interface and the inertia of the interfacial liquid F_{iner}:

$$F_f == -\eta_f \left(\frac{dv_f}{dz}\right)_{z=0} + F_{iner}$$

(28)

The first right hand term of equation (28) is given by equation (17) while the inertia force can be represented by [3]:

$$F_{iner} = nl_a \frac{\partial v_f(z=0)}{\partial t} = i\omega\rho_f l_a A_f$$

(29)

Where l_a is the inertia length and ρ_f is the density of fluid. Note that in reference [3], the value of l_a is given ≈ 4 Å and mentioned that this value is not sensitive to the bond strength.

So, replacing equation (29) in (28) gives:

$$F_f = (\alpha\eta_f + i\omega\rho_f l_a)A_f$$

(30)

Considering equation (18) and substituting equation (30) in (3) gives:

$$Z_L^s = \frac{A_f(\alpha\eta_f + i\omega\rho_f l_a)}{i\omega A_s(1+e^{2\alpha_s w})}$$

(31)

Based on equation (23) we have:

$$Z_L^s = \frac{i\omega A_s e^{-\alpha b}(1+e^{2\alpha_s w})(\alpha\eta_f + i\omega\rho_f l_a)}{i\omega A_s(1+e^{2\alpha_s w})} = (\alpha\eta_f + i\omega\rho_f l_a)e^{-\alpha b}$$

(32)

Considering Taylor series, we have:

$$1 + \alpha b = \frac{Z_L^s}{Z_L^{ns} + i\omega\rho_f l_a}$$

(33)

Considering $\alpha = (1-i)/\delta$ and $\delta = \sqrt{2\eta_f/\omega\rho_f}$ gives:

$$\frac{b}{\delta} = (1+i)\frac{(Z_L^s - Z_L^{ns} - i\omega\rho_f l_a)}{2(Z_L^{ns} + i\omega\rho_f l_a)}$$

(34)

As in the QCM, the frequency shift and damping rate are measured directly, using equations (1) and (20), the ratio between slip length and penetration length can be obtained from equation (34) and consequently slip length can be calculated.

3. Comparison with models in literature

Even though there are no independent studies available for dynamic slip (slip in flow conditions) measurement using vibrating solids in literature, we have compared the equations applied for static slip in the literature [2,3] by: 1) extending the use of slip equations to dynamic measurements and comparing them with our developed model and 2) computing static slips from our developed model and comparing it with static slip obtained from the models in literature.

3.1 Extending the use of slip equations in literature to compute dynamic slip and subsequent comparison with our model:

In this section, the proposed equations (results in main article) are compared with the methods presented by [2,3] (following equations) to calculate the slip length.

$$1 + 2\frac{b}{\delta} = -\frac{\frac{\Delta D}{2\pi}}{\frac{\Delta f}{f_0}}$$

(35)

$$\frac{1+\left[\left(1+2\frac{l_a}{\delta}\right)^2+1\right]\frac{b}{\delta}}{1+2\frac{l_a}{\delta}} = -\frac{\frac{\Delta D}{2\pi}}{\frac{\Delta f}{f_0}}$$

(36)

$$1 - 2\frac{b}{\delta} = -\frac{\frac{\pi\Delta f}{f_0}}{\sqrt{\frac{\omega\rho_f\eta_f}{2\rho_s\mu_s}}}$$

(37)

$$1 - 2\frac{b}{\delta} = -\frac{\frac{\pi|\Delta f|}{f_0}}{\sqrt{\frac{\omega\rho_f\eta_f}{2\rho_s\mu_s}}}$$

(38)

$$Z_L^{slip} = \sqrt{\frac{\omega\rho_f\eta_f}{2}}\left[1 + i(1 - 2\frac{b}{\delta})\right]$$

(39)

As can be seen from Fig. S4., considering the fact in [2], the positive value for b under the surface is considered in [2], the trend of slip versus flow rate is similar to the real value of our proposed equation. However, the equation proposed in [3] shows different behaviour (Fig. S5.) which can be explained by their assumption which considered stationary liquid which is no valid for the dynamics flow.

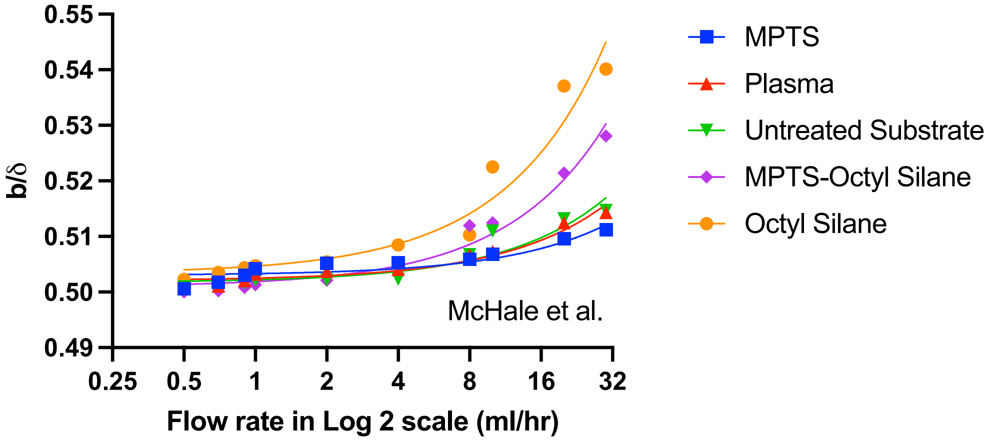

Fig. S4. Normalized slip length (real) as calculated using reference 2, plotted against flow rate.

The main advantage of our proposed equations in comparison with equations (35)-(39) is considering dissipation, hydrodynamics effect as well as the contribution of first water layer at the solid interface in calculation the liquid slip length which make our proposed approach more comprehensive.

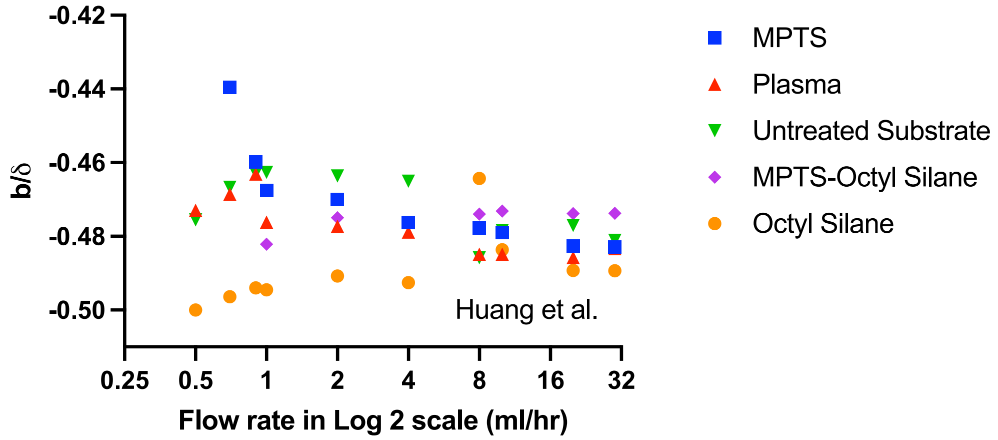

Fig. S5. *Normalized slip length (real) as calculated using reference 3, plotted against flow rate.*

3.2 Validation of our model with work in literature for slip in static liquid conditions only

We have verified our developed model and experiments with 3 different models proposed in the literature (called McHale, Heyward and Ellis in our paper, referenced accordingly). Note that all these models have measured slip in static mode using QCM surfaces and our model is the first one which measures slip in dynamic conditions with insights into inertial length of the system. In addition, none of the systems in the literature use surface modifications similar to our functionalization.

To validate our proposed equations, we have compared our equations with models in literature for the case of water resting on the QCM surface without flowing conditions, as well as ethanol and different concentration of glycerol for measurement of QCM response from different types of QCMs (with 5 MHz and 9 MHz as their natural frequencies). Note that the frequency of resonance in our work is 10 MHz. We have extracted the data from these QCMs works and used it to calculate slip using 3 models (where appropriate) and compared it with our method. The 3 models used for validation are listed below:

1. [Model 1] **McHale,** Glen, and M. I. Newton. "Surface roughness and interfacial slip boundary condition for quartz crystal microbalances." Journal of Applied Physics 95.1 (2004): 373-380. *This is reference 2 in the list of references*
2. [Model 2] G.L. **Heyward** and M. Thompson. “A transverse shear model of a piezoelectric chemical sensor.” Journal of Applied Physics 83.4 (1998): 2194-2201 *This is reference 4 in the list of references*

3. [Model 3] S. **Ellis**, and G. L. Hayward, Interfacial slip on a transverse-shear mode acoustic wave device, Journal of Applied Physics 94.12 (2003): 7856-7867. *This is reference 5 in the list of references*

A. Comparison with the work of McHale [model 1] where 10 MHz QCM was used.

Here, static slip of water on gold surface of the QCM is considered and we calculate the normalized slip length and compare the result with McHale [2]. Please note that we have used both dissipation and frequency shifts of the vibrating solid (QCM) to calculate the slip length (unlike McHale which does not consider dissipation effects). The Fig. S6. shows the comparison of slip lengths. Here in the Fig. S6. a) we show the frequency shifts and dissipation measured by our experiments. Fig. S6. b) compares the real value of the slip length in our model with the McHale's method.

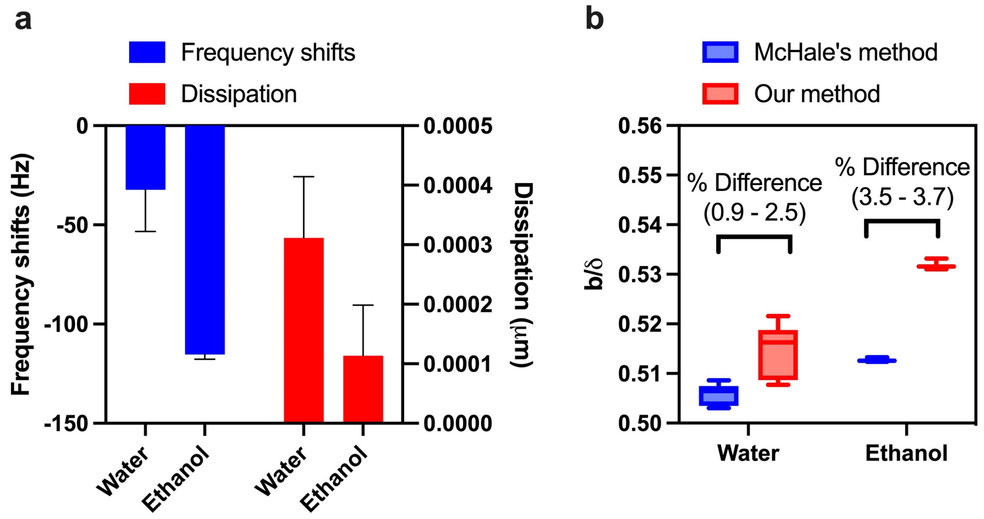

Fig. S6. *a) Frequency and dissipation measured in our experiments b) comparison of slip length calculated from our experimental data with the slip length calculated by McHale. Note that the error bars represent standard deviations in both subfigures.*

We can clearly see that the slip for ethanol is higher than that for water, which is in agreement with the results in McHale's work [2] and Ellis [5].

Additionally our calculated slip length has less than 3.7% difference for ethanol and less than 2.5% for water compared to McHale's work. Note that measurement using ethanol is non-trivial as ethanol is highly volatile and evaporates very quickly, which leads to more differences from measurement to measurement.

B. Our equations tested with data generated by Zhuang et al. [6] from 5 MHz QCM and compared with McHale's equation [2].

Here, we have extracted the frequency shifts from the work of Zhuang et al. [6] for different concentrations of glycerol, see Fig. S7. a) below for average frequency shifts in their work. We have then used these frequency shifts to compare the McHale and our method by calculating the slip length. Interestingly, we see that McHale's work and our method have less than 2% difference for concentrations of glycerol above 90%.

Below 90% concentration of glycerol, we had a maximum of 8.4% difference which suggests that as the solution becomes less viscous, dissipation and inertial effects are apparently easy to resolve using our developed equations. More studies are required to validate this relatively large difference (< 2% compared to < 8.4%) at lower concentrations of glycerol and here we just provide this extra information to elucidate differences.

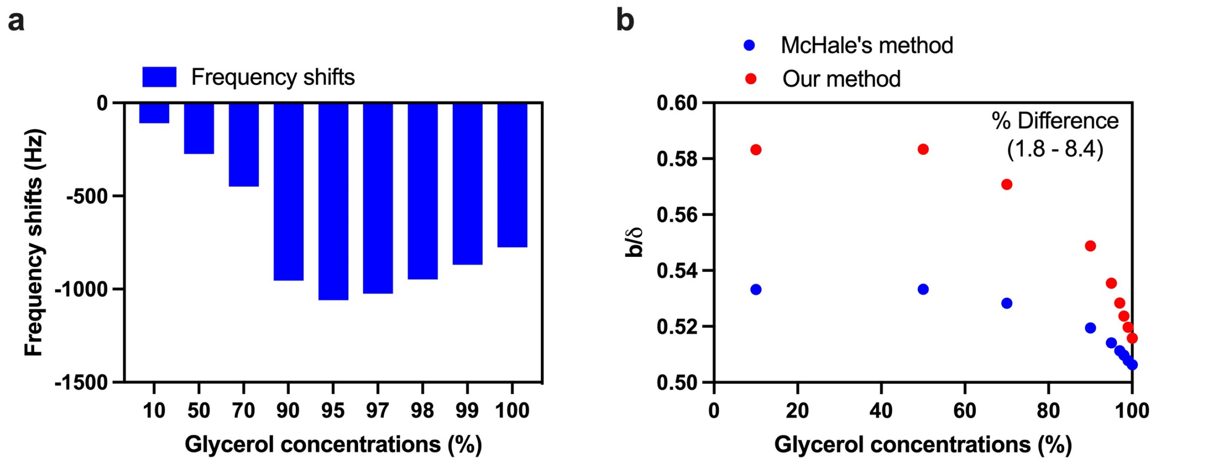

Fig. S7. *a) Frequency and dissipation measured by Zhuang et al. [6] b) comparison of slip length calculated from our experimental data with the slip length calculated by McHale using data in the work of Zhuang et al. [6].*

C. <u>Our equations compared with work of Heyward [5] from 9 MHz QCM and compared with McHale's equation[2].</u>

We have used the frequency shifts obtained by Heyward et al. for change in concentration of glycerol from 0-20% on a 9 MHz QCM, see Fig. S8. We then compared the changes in the magnitude of slip obtained by our equations and Heyward et al.. The maximum difference is less than 4.8% which is again due to consideration of dissipative effects in our equation.

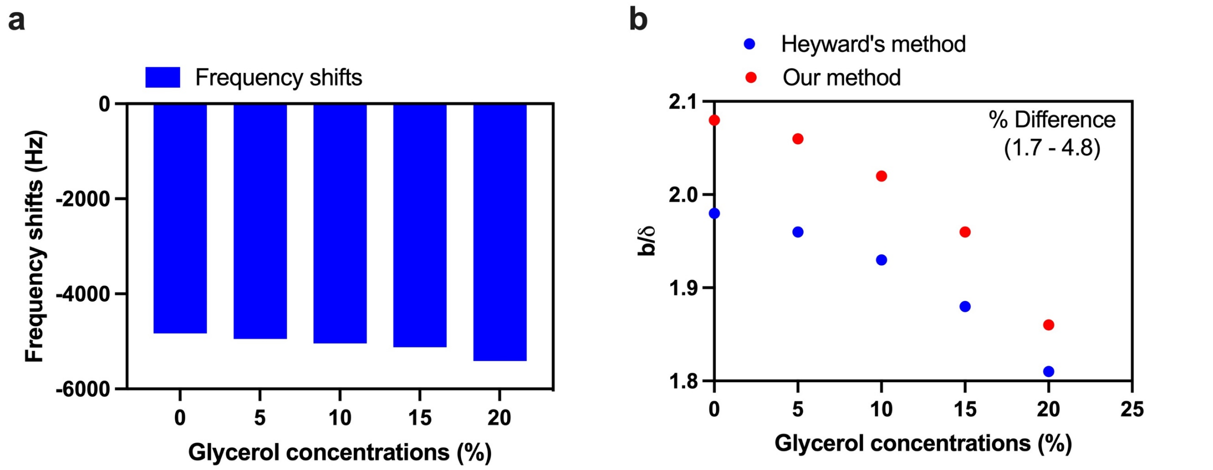

Fig. S8. *a) Frequency and dissipation measured by Heyward et al. [4] b) comparison of slip length calculated from our experimental data with the slip length calculated by Heyward's method.*

D. Direct comparison of slip length calculated by Ellis [5] using 9 MHz QCM

In **Fig. S9.** below we also compare our method with Ellis's method where the authors have calculated the slip length for ethanol without consideration of dissipation effects.

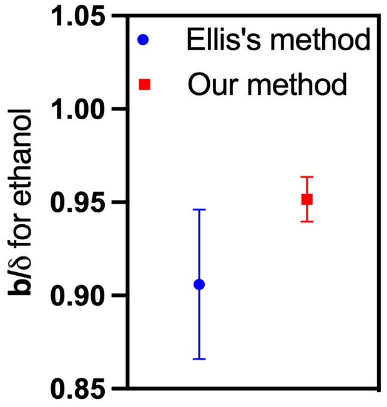

Fig. S9. *Comparison of normalized slip length calculated with Ellis's method. Our method shows less difference in slip length (from one experiment to another) in comparison to Ellis's method. Note that the error bars represent standard deviations.*

All the above comparisons serve as independent validation of slip length calculated by our model. From the comparisons we can conclude not only our model is valid in different conditions including different QCM frequencies and solution concentration, but also due to consideration of the effect of dissipation and inertial length, our method is more accurate than the well-known established QCM equations to calculate the slip length in static conditions.

4. Temperature variations during flow

The temperature in our experiments was measured using the in-built temperature sensor within the QCM measurement unit of OpenQCM instrument. The temperature changes in 6 different sensors with a flow rate of 30 ml/hr are observed to be less than 0.6 °C, see Fig. S10. The base line temperature is 25 $\pm$ 1 °C. This suggests that there is less than 1.5% change in temperature (on average) at the highest flow rate tested in our experiment (i.e. 30 ml/hr) and therefore the temperature effects are negligible in our experiments performed to validate the developed analytical model.

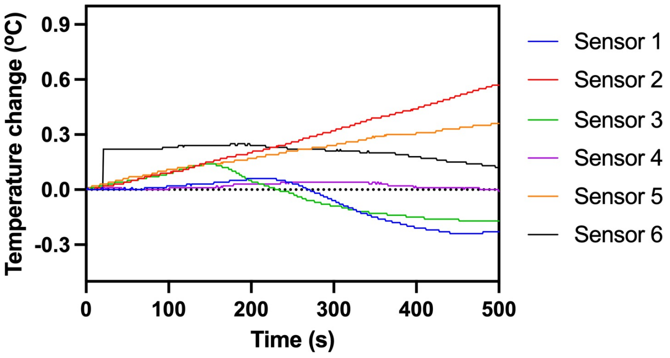

Fig. S10. *Temperature change of QCM sensor when exposed to flow rate of 30 ml/hr for a duration of 500 seconds.*

5. FTIR

In Fig. S11. we share the Fourier Transform Infrared Spectroscopy (FTIR) characterization of our surface modification. Typical chemical bonds which appear for different chemical used in surface modifications are also discussed in main text of the manuscript.

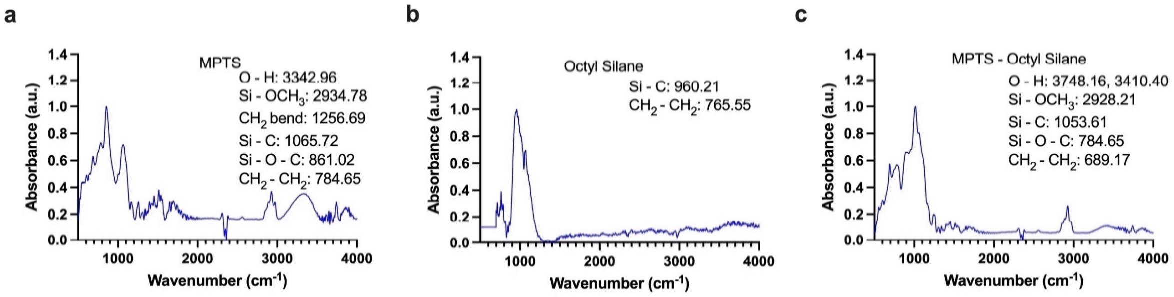

Fig. S11. *a-c) Correspond to Fourier Transform Infrared Spectroscopy (FT-IR) of MPTS, Octyl Silane and MPSTS-Octyl Silane treated surfaces respectively.*

We have performed the FTIR at least 16 times on 3 different QCM substrates for each condition over the whole surface before and after flowing the water. We see no aging effects in our FTIR before and after the experiment which suggests that our surface modification was robust to consider experimental data suitable for analytical modelling of slippage, see Fig. S12.

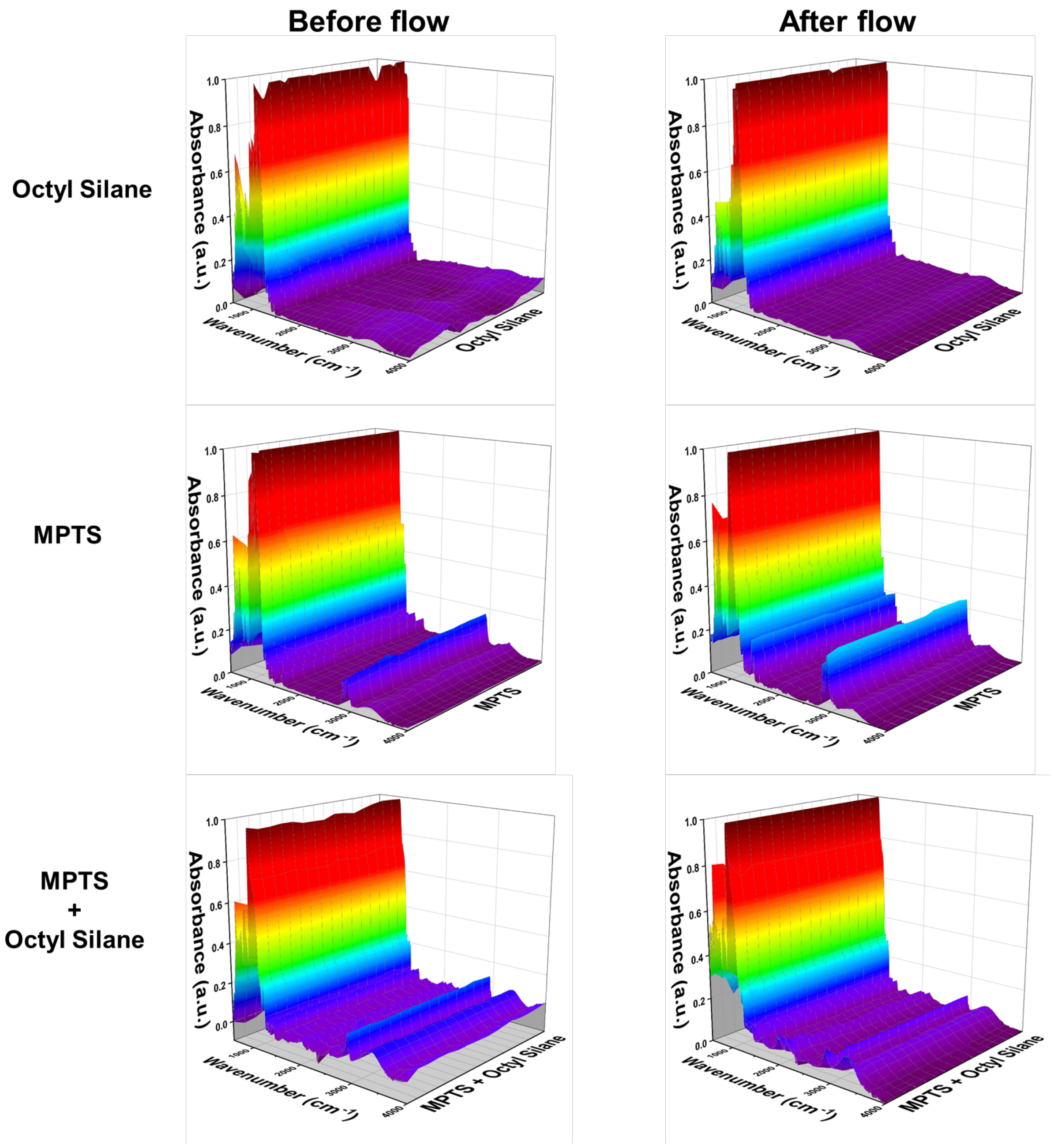

Fig. S12. *FTIR of functionalized QCM surfaces before and after completion of the flow experiments.*

6. AFM characterization

We have performed AFM characterization to check the roughness of the surface before and after the completion of the flow experiments, see Fig. S13. These images were acquired using Park systems XE-100 in non-contract mode at a scan rate of 0.3 Hz. The size of the images are 50 μm x 50 μm.

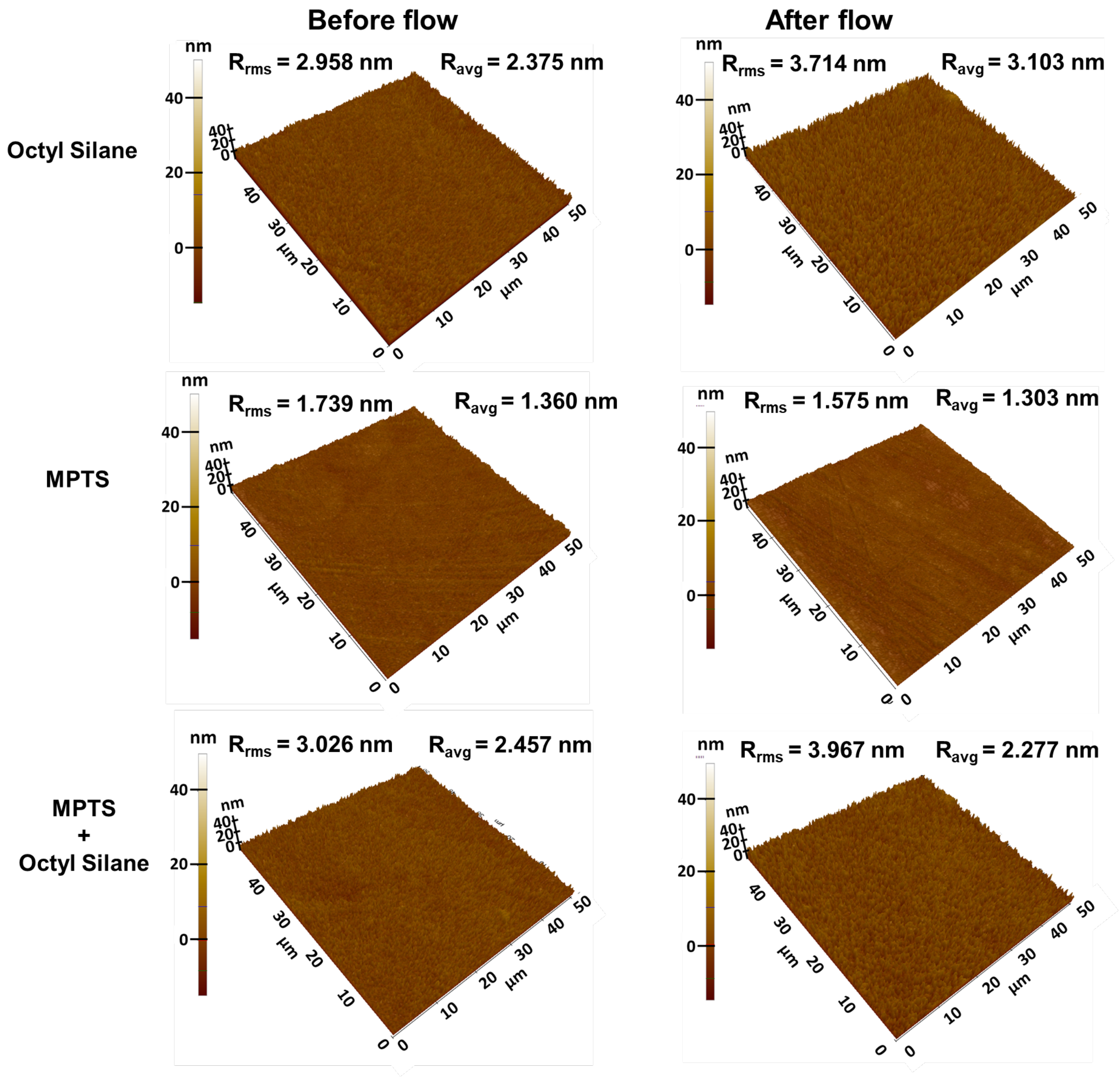

Fig. S13. *AFM of the functionalized QCM surfaces before and after completion of the flow experiments.*

Supplementary References

1. Meléndez, M., Vázquez-Quesada, A. & Delgado-Buscalioni, R. Load impedance of immersed layers on the quartz crystal microbalance: a comparison with colloidal suspensions of spheres. *Langmuir* 36, 9225-9234 (2020).
2. McHale, G. & Newton, M. I. Surface roughness and interfacial slip boundary condition for quartz crystal microbalances. *Journal of Applied Physics,* **95** 372-380 (2004).
3. Huang, K. & Szlufarska, I. Friction and slip at the solid/liquid interface in vibrational systems." *Langmuir* **28**, 17302-17312 (2012).
4. Heyward, G.L. & Thompson, M. A transverse shear model of a piezoelectric chemical sensor. *Journal of Applied Physics* **83,** 2194-2201 (1998).
5. Ellis, S. & Hayward, G.L. Interfacial slip on a transverse-shear mode acoustic wave device, *Journal of Applied Physics* **94**, 7856-7867 (2003).
6. Zhuang, H., Lu, P.,Lim, S.P. & Lee, H.P. Effects of Interface Slip and Viscoelasticity on the Dynamic Response of Droplet Quartz Crystal Microbalances, *Analytical Chemistry* **80**, 7347–7353 (2008).
